# Unveiling individuality in the early phase of motor learning: a machine learning approach for analysing weightlifting technique in novices

**DOI:** 10.3389/fbioe.2024.1426058

**Published:** 2024-07-30

**Authors:** Achraf Ammar, Marvin Leonard Simak, Atef Salem, Fabian Horst, Wolfgang Immanuel Schöllhorn

**Affiliations:** ^1^ Department of Training and Movement Science, Institute of Sport Science, Johannes Gutenberg-University Mainz, Mainz, Germany; ^2^ Research Laboratory, Molecular Bases of Human Pathology, LR19ES13, Faculty of Medicine of Sfax, University of Sfax, Sfax, Tunisia; ^3^ High Institute of Sport and Physical Education of Sfax, University of Sfax, Sfax, Tunisia

**Keywords:** kinematics, kinetics, support vector machines, individuality, whole-body movement, strength, coordination, barbell

## Abstract

**Introduction:**

Despite the growing body of evidence highlighting the individuality in movement techniques, predominant models of motor learning, particularly during the acquisition phase, continue to emphasise generalised, person-independent approaches. Biomechanical studies, coupled with machine learning approaches, have demonstrated the uniqueness of movement techniques exhibited by individuals. However, this evidence predominantly pertains to already stabilised movement techniques, particularly evident in cyclic daily activities such as walking, running, or cycling, as well as in expert-level sports movements. This study aims to evaluate the hypothesis of individuality in whole-body movements necessitating intricate coordination and strength among novice participants at the very beginning of an acquisition phase.

**Methods:**

In a within-subject design, sixteen highly active male participants (mean age: 23.1 ± 2.1 years), all absolute novices in the learning task (i.e., power snatch of Olympic weightlifting), participated in randomised snatch learning bouts. These bouts comprised 36 trials across various motor learning models: differential learning contextual interference (serial, sCIL; and blocked, bCIL), and repetitive learning. Kinematic and kinetic data were collected from three standardised snatch trials performed following each motor learning model bout. The time-continuous data were input to a linear Support Vector Machine (SVM). We conducted analyses on two classification tasks: participant and motor learning model.

**Results:**

The Support Vector Machine classification revealed a notably superior participant classification compared to the motor learning model classification, with an averaged prediction accuracy of 78% (in average ≈35 out of 45 test trials across the folds) *versus* 27.3% (in average ≈9 out of 36 test trials across the folds). In specific fold and input combinations, accuracies of 91% *versus* 38% were respectively achieved.

**Discussion:**

Methodically, the crucial role of selecting appropriate data pre-processing methods and identifying the optimal combinations of SVM data inputs is discussed in the context of future research. Our findings provide initial support for a dominance of individuality over motor learning models in movement techniques during the early phase of acquisition in Olympic weightlifting power snatch.

## 1 Introduction

Motor learning encompasses the acquisition, stabilisation, and refinement of movement techniques. Historically, prevailing motor learning models have emerged from three primary research domains, each with a different focus: training science (refinement), sports pedagogy (acquisition), and sports psychology (stabilisation) (Newell, 1990; Starkes and Ericsson, 2003; Naul and Scheuer, 2020; Schöllhorn et al., 2022). While research approaches in training science and sports pedagogy are typically idiographic and learner-centred, those in sports psychology are often characterised as nomothetic and group-oriented. The stronger focus on the individual learner on the part of sports pedagogy was reflected in practice, among other things, in the development of methodical exercise and game series (Gaulhofer and Streicher, 1924; Mester, 1959; Mahlo, 1965), in which the initial difficulty level of the exercise or game is individually adapted to the learner’s ability level from the outset, but nonetheless with an orientation towards a person-independent role model at the end of the learning process. In training science individuality was ascribed a specific role within the framework of the “principle of individuality” Matveev (1966). The principle states that every training intervention must take into account the specific needs and abilities of the individuals for whom it is designed, regardless of the motor learning and/or training phase. Since training in sports is not only aimed at endurance, strength, agility, or tactics, but also at movement techniques and coordination, it has a large overlap with the research subject of motor learning of sports movements. For the acquisition process of a movement technique most intriguing, the “principle of individuality” was modified by Harre (1975) to the “principle of increasing individualisation” that was closely related with the “principle of increasing specialisation” (Schöllhorn et al., 2006). Moreover, its importances was reduced by shifting it from the first to eighth position. This principle constrained the individuality of training measures to the last end of the training process, to the highest performance level. This meant that up to a certain performance level all athletes had to exercise the same. Only specialised and high-performance athletes received individual training contents and schedules. While the “principle of individuality” assumes the uniqueness of an athlete as fundamental and independent of time, the “principle of increasing individualisation” considers the uniqueness as an add on to a general technique that only develops at the highest level of performance. Both training principles are associated with different practical consequences. Although individuality in novices and advanced athletes was still lacking, the necessity of individuality at the absolute top level had long been suspected in the form of these preset training principles.

In comparison, prevailing motor learning models offered by sport psychology such as repetitive learning (RL), variability of practice learning (VPL), and contextual interference learning (CIL) have operated for long under the assumption of a widely person-independent standardised movement technique. The RL, VPL, and CIL models emphasise the importance of imitating a generalised technique role model and engaging in correct imitations through repeated executions to improve the learner’s proficiency in performing a specific motor skill with greater accuracy and precision (Schöllhorn et al., 2022).

A growing number of biomechanical studies indicate the uniqueness of individual movement techniques (i.e., which indicates that the characteristic should not be the same for any two persons) calling into question the orientation and imitation towards general technique role models in motor learning (Bauer and Schöllhorn, 1997; Schöllhorn and Bauer, 1997; Horst et al., 2017b; Horst et al., 2023b). Aiming at coping with the orientation towards general technique role models, the differential learning (DL) model suggests that motor learning should avoid the imitation of such general role models throughout the learning process (i.e., in acquisition, stabilisation, and refinement phase). Instead, DL advocates embracing a diverse range of executed variants of movement techniques during motor learning and variations from one execution to another (Schöllhorn et al., 2006; Schöllhorn et al., 2009). The variation of movement technique from execution to execution should be tailored to the learner’s individual and situational characteristics within an adaptive stochastic resonance process throughout a learning intervention (Schöllhorn, 2000; Schöllhorn and Horst, 2019; Apidogo et al., 2021; Apidogo et al., 2022; Schöllhorn et al., 2022; Apidogo et al., 2023).

From a general perspective, motor learning interventions can be regarded as external manipulations of movement techniques. This prompts inquiry into the degree to which such interventions influence the movement techniques of individuals, raising questions about the persistence of individuality in movement techniques in the presence of internal or external perturbations.

The persistence of individual movement techniques over time (i.e., which means that the characteristic should be invariant with time) was first indicated through machine learning-based analysis of biomechanical data of movement techniques of world-class male discus throwers for 1 year (Bauer and Schöllhorn, 1997) and world-class female javelin throwers over a period of 5–6 years (Schöllhorn and Bauer, 1998). Studies on running (Schöllhorn and Bauer, 1998a), sprinting (Schöllhorn et al., 2001), and walking (Horst et al., 2016; Horst et al., 2017b) indicated similar findings. The persistence of individuality against internal and external perturbations was also investigated. For instance, emotions (Janssen et al., 2008), fatigue (Janssen et al., 2011; Burdack et al., 2020), music (Janssen et al., 2008), shoe heel heights (Schöllhorn et al., 2002a), orthopaedic insoles (Schöllhorn et al., 2002b), or running shoes (Horst et al., 2023a) supported the predominance of individuality within a single movement technique. Two recent studies address individuality across multiple movement techniques indicated that movement techniques overlap individuality in sport techniques, e.g., shot put, discus, and javelin throwing (Horst et al., 2020) and in everyday movements, e.g., walking, running, and handwriting (Burdack et al., 2023).

Studies on individuality so far predominantly focused on samples that either had stabilised the respective movements through a corresponding number of repetitions (e.g., gait) (Horst et al., 2016; Horst et al., 2017b; Horst et al., 2023b), showed extreme performances close to an individual’s maximal potential (e.g., javelin throwing) (Schöllhorn Bauer, 1998a)), or both (e.g., extreme shoe heel heights) (Schöllhorn et al., 2002a). Correspondingly the subjects were already in the stabilisation or refinement phase of a learning process or reached a performance limit where individual characteristics become more expressed (Schöllhorn et al., 2002a). The hypothesis of individuality is therefore supported at a higher and highest automatisation level in movement techniques in high-performance sports (i.e., world-class athletes) or movement techniques of daily life (e.g., walking), but not for novices. The observed individuality of movement techniques (after the acquisition phase) can be the result of three different conditions (Horst et al., 2020). First, despite identical initial movement techniques, the individuality of movement techniques is the result of varying persistence to motor learning interventions. Second, individuality of movement techniques exists before the acquisition phase and is only minimally influenced by motor learning interventions. Third, both individuality of movement techniques exists before the acquisition phase and varying persistence to motor learning interventions (Horst et al., 2020).

Each of the three conditions is based on the initial state of individuality or non-individuality of movement techniques prior to the acquisition phase, which has so far been largely unexplored. To disentangle the influence of external interventions through motor learning models and individual movement techniques, it is crucial to assess individual movement techniques not only at the end of a stabilisation process but also in the early stages of the acquisition process. Furthermore, the majority of the available studies have primarily evaluated the individuality hypothesis in movement techniques that were dominated by coordination with limited emphasis on a possible interaction of strength and coordination like in whole-body weightlifting movement techniques. Moreover, the risk of injury increases particularly when lifting weights dynamically, as force peaks can occur in directions that beginners are unable to compensate for, especially when they deviate significantly from their individual strength patterns. Therefore, identifying individual movement techniques among novice weightlifters is beneficial for developing healthy (Kumaran et al., 2022) and effective (Rushall, 1979; Bompa, 1983; Rushall and Pyke, 1990) training and learning interventions. This area of research has the potential to revolutionize training methods for novice weightlifters, making a significant contribution to sports science and biomechanics. To build upon this understanding, this study aims to identify eventual individual weightlifting techniques and evaluate the persistence of the individual weightlifting techniques across four single bouts, each of four different motor learning models, during the early acquisition phase in absolute novices. Specifically, time-continuous biomechanical data from three standardised snatch executions after the learning bout will be subject to machine learning classification techniques, distinguishing between “individual” and “motor-learning” short-term effects, for the classification and prediction of snatch movement technique. We hypothesise that the employed machine learning method (i.e., support vector machine) will achieve a higher prediction accuracy in the participant-based classification, thereby providing initial empirical support for the individuality hypothesis in the context of single bouts of various motor learning models applied to weightlifting acquisition in absolute novices.

## 2 Materials and methods

### 2.1 Participants

Sixteen highly active male participants (age: 23.1 ± 2.1 years, body mass index: 24.1 ± 2.2 kg/m2), were recruited for this study. None of the participants had prior experience with the to be learned skill, the power snatch. After the protocol, potential risks, and study benefits were presented, participants provided written consent to participate in the study. The inclusion criteria for participants were as follows: aged between 18 and 29 years, male, and at least 2 years of experience in fitness and/or CrossFit club (i.e., including at least 6 months of performing barbell-based exercises). Participants with prior involvement in Olympic weightlifting, current or past neurological and/or cardiovascular issues, eye disorders, psychiatric conditions, orthopaedic ailments, muscular disorders, and those taking medications that could impact the cardiovascular system were excluded based on the criteria. Furthermore, all participants had no chronic diseases or sleep disturbances. During the experimental period, participants reported experiencing good to very good sleep quality, alongside maintaining a very active lifestyle. This was demonstrated by an average of more than 2 h of physical activity per weekday, which included walking as well as moderate- and vigorous-intensity activities. Participants were not engaged in napping during the experimental period. The study was conducted according to the Declaration of Helsinki and approved by the local ethics committee of Faculty 02: Social Sciences, Media, and Sport at Johannes Gutenberg-University of Mainz. Written informed consent was obtained from all participants who were naive to the purpose of the study and were coded with numbers for the anonymity of personal data.

### 2.2 Experimental design

In a randomised within-subject design, each of the 16 participants performed single bouts of four different motor learning models, namely, RL, CIL in its blocked (bCIL), which corresponds to VPL, and serial form (sCIL), and DL. After a familiarisation session, participants visited the laboratory on four separate occasions, with at least a 1-week washout period in between. During each test session, a single motor learning model was implemented, all in a randomised order, involving a single training bout. Each bout comprised 36 trials of power-snatch derivatives, according to one of the four tested motor learning models with a 3-min standardised duration (Ammar et al., 2024a; Ammar et al., 2025). Each training bout utilised the same empty barbell weighing 10 kg. All testing sessions were conducted in the afternoon, as previously suggested by Ammar et al. (2015a); Ammar et al. (2015b); Ammar et al. (2016), to minimise the effect of diurnal biological variations (Ammar et al., 2017; Ammar et al., 2018a). The measurements were conducted in a laboratory setting, with standardised and minimised changes in brightness and temperature. 5 min following each bout, three 20 kg barbell power snatch trials were performed, without any instruction, and barbell kinematics and kinetics data were collected. Participants were instructed to wear identical shoes during all test sessions.

### 2.3 Motor learning models

The training protocol for the RL model comprised 36 sets of power snatch trials. In the case of the two CIL models (bCIL and sCIL), the training protocols incorporated not only the power snatch but also two variations: the high pull snatch (Waller et al., 2009) and the snatch power jerk (Soriano et al., 2019). These three techniques were practised either in blocked (bCIL) or serial (sCIL) order. Because of the different relative timings and different sequences of muscle contractions of all three techniques, CIL is considered to switch between different general motor programs. The DL model incorporated the practice of these three techniques in a serial order but each technique with additional movement variations. Variations in foot starting position, barbell starting position, final positions, practising with eyes closed, and utilising an unstable surface were incorporated. Further details on each motor learning protocol, including the movement schedule and resting intervals, are elaborated in Ammar et al. (2024a); Ammar et al. (2025).

### 2.4 Measurements

#### 2.4.1 Data acquisition

The power snatch trials were performed after each training bout on a 2.4 × 0.9 m weightlifting platform and were recorded using Qualisys Track Manager 2023.2 (Qualisys AB, Sweden). The 3D barbell positions were measured using nine synchronised, commercially available infrared cameras (Type Oqus 300/310+; 250 Hz; Qualisys AB, Sweden) positioned around the platform at a distance of approximately 6 m from the lifting area. Two reflective markers were attached to the right and left ends of the barbell. The calibration was executed before each test session using a carbon fibre calibration kit, including a 500 mm wand and an L-frame with reflective markers. In addition, the 3D ground reaction forces (GRFs) were measured using two Kistler force platforms (Type 9287CA; 1,000 Hz; Kistler, Switzerland) embedded in the ground. During the power snatch trials, the tested participants positioned one foot each on a force platform. Before the three dynamic power snatch trials, a static measurement (without barbell) was performed to calculate the participant’s body weight.

#### 2.4.2 Data processing

**FIGURE 1:**
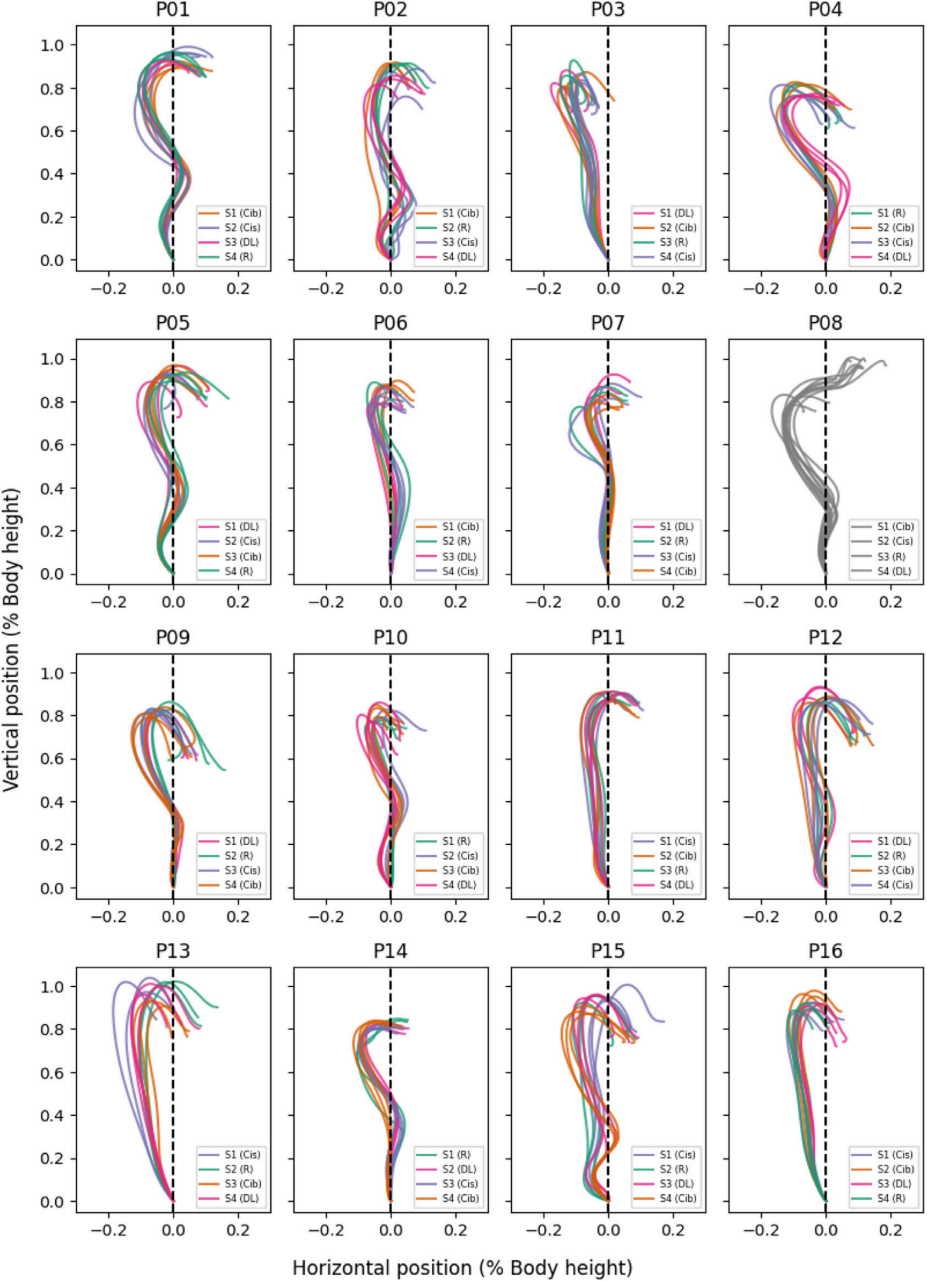
Processed position trajectories of the barbell during the power snatch of the 16 participants (P01 to P16). Each subfigure shows the anterior-posterior displacement of the barbell along the abscissa (x-axis) and the vertical displacement of the barbell along the ordinate (y-axis) as a multiple of the participant’s body height. The randomization of the motor learning models within the 4 test sessions (S1 to S4) for each participant is detailed in the legend of each subfigure. Please note that the subfigure labelled P08 represents the trajectories excluded from the analysis, belonging to participant 08.

For the kinematic analysis, the mean position of the reflective markers attached to the right and left end of the barbell was calculated to provide the trajectory of the barbell centre and reduce the induction of artefacts associated with asymmetrical movement execution (Ammar et al., 2018b; Ammar et al., 2019). For the kinetic analysis, the total GRF was calculated by summing the recorded force vectors of both platforms. The data was filtered using a fourth-order Butterworth low-pass filter with a cut-off of 4 Hz for the barbell position trajectories and 15 Hz for the GRF trajectories. The barbell velocity was calculated by numerical estimation based on the filtered barbell position trajectory. All processed trajectories were trimmed to the movement phase from the start of the movement to the catch position (Nago et al., 2019). The start position was defined as the time when the vertical barbell velocity was ≥0.01 m∙s^-1^ and the catch position was defined as the first instance at which the barbell reached a vertical velocity of 0 m∙s^−1^ after the phase of negative vertical velocity following the maximum vertical displacement (Nagao et al., 2019; Ammar et al., 2024a; Ammar et al., 2025). For one of the 16 participants recruited (participant 08), the catch phase could not be identified using the definitions described by Nagao et al. (2019). Data related to this participant was therefore excluded from further analysis (Figure 1). The trimmed barbell position and velocity trajectories were normalised based on body height, and the GRF on the basis of body mass. All trajectories were time normalised by linearly interpolating the trajectories to 101 time points (0%–100% of the power snatch movement). Finally, the initial values were subtracted from the barbell position trajectories to standardise them, ensuring that all trajectories start from a baseline value of zero. Figure 1 illustrates the processed barbell position trajectories in the sagittal plane for the 16 participants.

#### 2.4.3 Data analysis (machine learning-based classification)

In total, the kinematic (i.e., position and velocity) and kinetic (i.e., ground reaction force) trajectories of 180 power snatch lifts performed by 15 participants after each of 4 training bouts formed the basis for the machine learning-based classification analysis. Two classification tasks were investigated: participant classification and motor learning model classification. Support Vector Machines (SVMs) (Cortes and Vapnik, 1995) were used as a machine learning classification method, as they have shown competitive performance in the classification of biomechanical data (Horst et al., 2019; Burdack et al., 2020; Slijepcevic et al., 2021; Horst et al., 2023) and favourable runtime efficiency. We utilised the Liblinear Toolbox (version 1.4.1) with a linear kernel and an L2-regularised L2-loss function (Fan et al., 2008). The hyperparameter C was set to 1, a standard default that balances regularization and ensures good generalization performance (Fan et al., 2008).

**FIGURE 2:**
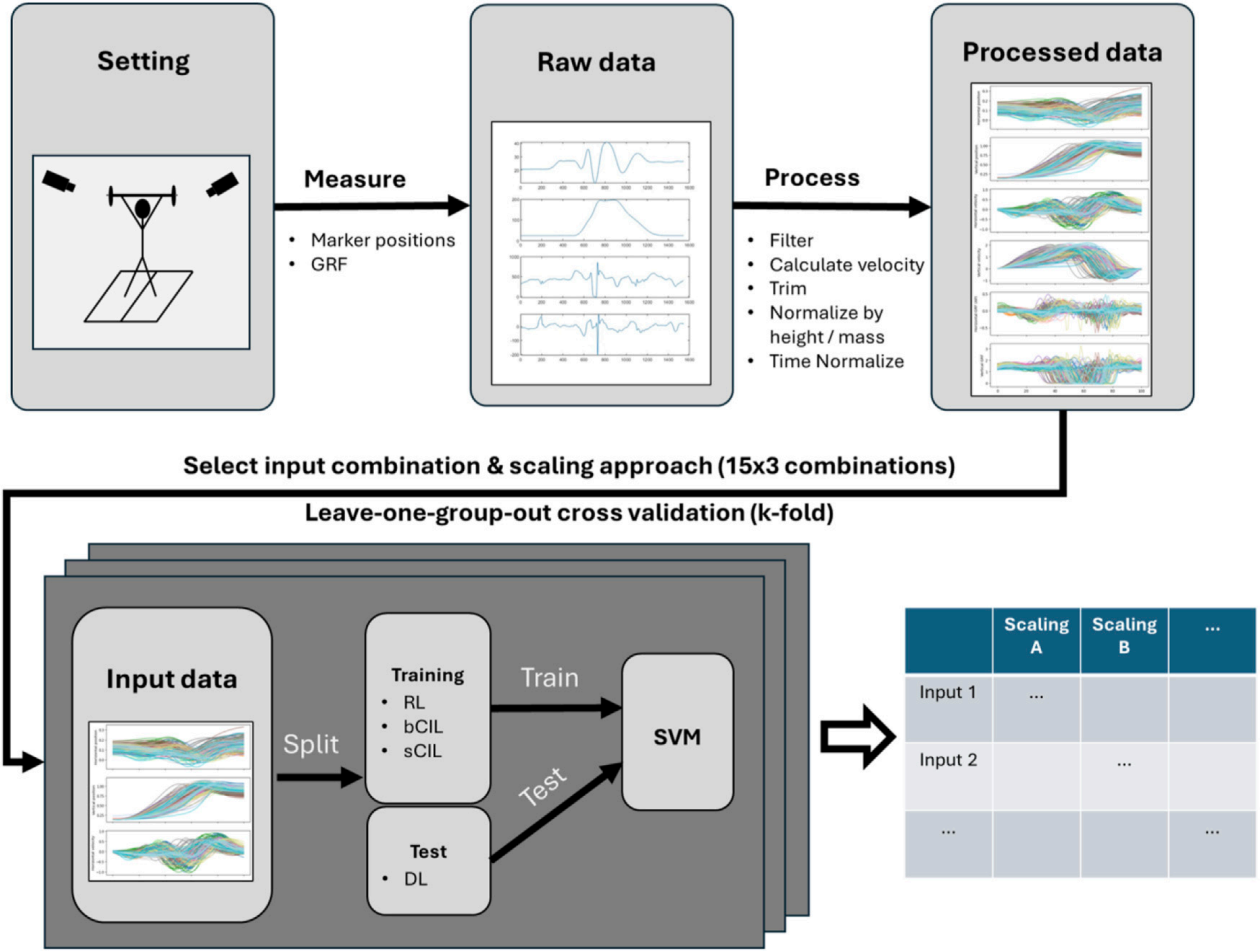
Machine learning-based classification procedures.

As Figure 2 shows, for both classification tasks, a leave-one-group-out cross-validation approach was utilised to evaluate the performance of the SVM models across motor learning models (in the participant classification) and participants (in the motor learning model classification). For participant classification, all trials from three out of four test conditions were used as training data (135 trials), while the trials from the remaining condition were used as test data (45 trials). This procedure was repeated so that the trials of each test condition were used once as test data, resulting in a 4-fold cross-validation (Fold 1: “bCIL” was tested, Fold 2: “sCIL” was tested, Fold 3: “DL” was tested, Fold 4: “RL” was tested). Similarly, a 5-fold cross-validation was performed for the classification of the test conditions, leaving out a subset of the participants in each case. This involved dividing the participants into 5 groups and repeatedly using one of the groups (36 trials) as validation of the model trained on the remaining groups (144 trials). This procedure resulted in a 5-fold cross-validation with participants “9, 15, 6” being tested in Fold 1, participants “1, 7, 10” being tested in Fold 2, participants “4, 2, 12” being tested in Fold 3, participants “14, 11, 3” being tested in Fold 4, and participants “5, 16, 13” being tested in Fold 5.

In the classification, we evaluated the performance of 15 combinations of trajectory-related input (position.AP/V, velocity.AP/V, GRF.AP/V), as detailed in the results tables. These combinations included 9 focusing solely on kinematic input, 3 on kinetic input, and 3 integrating both kinetic and kinematic inputs. This led to an examination of 15 input trajectory configurations alongside 3 scaling approaches, yielding a total of 45 distinct combinations. Regarding the scaling approaches this include 1. no scaling; 2. batch scaling with data being scaled based on the absolute maximum value in the training data (separately for each trajectory); and 3. instance scaling with data being scaled based on the absolute maximum value of the trial (separately for each trajectory).

To identify the input features utilised by SVM models in the given classification tasks, we employed Layer-wise relevance propagation (LRP) (Bach et al., 2015; Horst et al., 2019), a method widely used in explainable artificial intelligence designed to reveal the basis for machine learning model predictions. The LRP method allows for the decomposition of the SVM model’s predictions into relevance scores for each input feature, highlighting the contributions of specific features to the classification decisions (Bach et al., 2015; Horst et al., 2019). Particularly, these relevance scores indicate the input values used by the SVM model for its prediction, with positive scores supporting the classification prediction and negative scores opposing it. In this study, we decomposed ground truth class labels and analysed only positive input relevance scores (i.e., those favouring the ground truth label), which were subsequently normalised to the respective maximum of each trial. Following this, the relevance scores were aggregated across all trials to generate model-level explanations, providing insights into the trained machine learning model’s overall functionality. This approach facilitated the identification of task-specific prototypes and characteristic patterns by calculating the average LRP relevance scores across all trials and cross-validation folds in the test set.

The data processing and analysis was performed using Matlab R2023b (MathWorks, USA).

In Table 1 and Table 2 of the results section, averaged accuracies were introduced. These values were calculated using the “average” function of Excel which is based on the “arithmetic mean” formula: sum of all values (in all folds and scaling approaches) divided by the number of values.

**TABLE 1:** Prediction accuracy of the participant classification with leave-learning-model-out cross-validation for different data partitions (i.e., folds). In Fold 1 "bCIL " was tested, Fold 2 "sCIL” was tested, Fold 3 "DL” was tested, Fold 4 "RL” was tested.

Variables/accuracy	Scaling condition + Test/training data															Averaged accuracy
	No-scale					Instance scale					Batch scale					
	Fold 1	Fold 2	Fold 3	Fold 4	Mean ± SD	Fold 1	Fold 2	Fold 3	Fold 4	Mean ± SD	Fold 1	Fold 2	Fold 3	Fold 4	Mean ± SD	
Random Baseline	6.7	6.7	6.7	6.7	6.7	6.7	6.7	6.7	6.7	6.7	6.7	6.7	6.7	6.7	6.7	6.7
Barbell Kinematic																
Position.AP	42.2	37.8	35.6	51.1	41.7 ± 6.9	53.3	46.7	53.3	57.8	52.8 ± 5.8	46.7	46.7	44.4	62.2	50.0 ± 8.2	48.1 ± 7.8
Position.V	44.4	51.1	44.4	42.2	45.6 ± 3.8	42.2	48.9	46.7	42.2	45.0 ± 3.3	42.2	51.1	44.4	42.2	45.0 ± 4.2	45.2 ± 3.5
Position.AP, Position.V	77.8	66.7	60.0	75.6	70 ± 8.22	68.9	62.2	57.8	71.1	65.0 ± 3.12	75.6	64.4	62.2	84.4	71.77 ± 10.3	68.9 ± 8.1
Velocity.AP	55.6	46.7	53.3	62.2	54.4 ± 6.4	53.3	53.3	57.8	62.2	56.7 ± 4.3	57.8	46.7	53.3	62.2	55 ± 6.6	55.4 ± 5.4
Velocity.V	62.2	51.1	55.6	55.6	56.1 ± 4.6	64.4	48.9	57.8	55.6	56.7 ± 6.4	62.2	46.7	53.3	55.6	54.4 ± 6.4	55.7 ± 5.4
Velocity.AP, Velocity.V	80.0	66.7	64.4	86.7	74.4 ± 10.7	73.3	73.3	64.4	80.0	72.8 ± 6.4	82.2	64.4	66.7	86.7	75 ± 11.1	74.1 ± 8.8
Position.AP, Velocity.AP	60.0	48.9	53.3	64.4	56.7 ± 6.9	44.4	51.1	53.3	66.7	53.9 ± 9.3	64.4	53.3	55.6	71.1	61.7 ± 8.2	57.2 ± 8.0
Position.V, Velocity.V	77.8	68.9	60.0	64.4	67.8 ± 7.59	71.1	60.0	57.8	60.0	62.2 ± 6.0	77.8	68.9	55.6	66.7	67.2 ± 9.1	65.7 ± 7.4
Position.AP, Position.V, Velocity.AP, Velocity.V	88.9	75.6	73.3	88.9	81.1 ± 8.4	77.8	71.1	64.4	80.0	73.3 ± 7.0	91.1	71.1	66.7	86.7	78.9 ± 11.8	78.0 ± 9.2
Kinetic																
GRF.AP	42.2	37.8	40.0	44.4	41.1 ± 2.9	46.7	35.6	31.1	40.0	38.3 ± 6.6	44.4	35.6	42.2	46.7	42.2 ± 4.8	40.6 ± 4.8
GRF.V	55.6	53.3	42.2	51.1	50.6 ± 5.8	55.6	55.6	33.3	44.4	47.2 ± 10.6	60.0	51.1	33.3	44.4	47.2 ± 11.2	48.3 ± 8.8
GRF.AP, GRF.V	71.1	62.2	53.3	53.3	60 ± 8.5	71.1	44.4	55.6	44.4	53.9 ± 12.6	73.3	46.7	55.6	60.0	58.9 ± 11.1	57.6 ± 10.2
Kinematic + Kinetic																
Position.AP, Velocity.AP, GRF.AP	73.3	64.4	64.4	75.6	69.4 ± 5.8	64.4	51.1	46.7	68.9	57.8 ± 10.6	82.2	68.9	64.4	73.3	72.2 ± 7.6	66.5 ± 9.9
Position.V, Velocity.V, GRF.V	66.7	68.9	62.2	71.1	67.2 ± 3.8	68.9	73.3	60.0	68.9	67.8 ± 5.6	77.8	66.7	66.7	73.3	71.1 ± 5.4	68.7 ± 4.9
All: Position.AP, Position.V, Velocity.AP, Velocity.V, GRF.AP, GRF.V	88.9	80.0	66.7	77.8	78.3 ± 9.1	84.4	75.6	60.0	84.4	76.1 ± 11.5	91.1	77.8	66.7	86.7	80.6 ± 10.8	78.3 ± 9.7

**TABLE 2:** Prediction accuracy of the learning-model-classification with leave-participant-out cross-validation for different data partitions (i.e., folds). Fold 1: participants “9, 15, 6" were tested; Fold 2: participants “1, 7, 10" were tested; Fold 3: participants “4, 2, 12" were tested; Fold 4: participants “14,11,3" were tested; Fold 5: participants “5,16,13" were tested.

Variables/accuracy	Scaling condition + Test/training data																		Averaged accuracy
	No-scale						Instance scale						Batch scale						
	Fold 1	Fold 2	Fold 3	Fold 4	Fold 5	Mean ± SD	Fold 1	Fold 2	Fold 3	Fold 4	Fold 5	Mean ± SD	Fold 1	Fold 2	Fold 3	Fold 4	Fold 5	Mean ± SD	
Random Baseline	25	25	25	25	25	25	25	25	25	25	25	25	25	25	25	25	25	25	25
Barbell Kinematic																			
Position.AP	8.3	25.0	25.0	27.8	19.4	21.1 ± 7.8	16.7	25.0	25.0	30.6	19.4	23.3 ± 5.4	11.1	27.8	13.9	33.3	25.0	22.2 ± 9.4	22.2 ± 7.2
Position.V	16.7	22.2	30.6	19.4	16.7	21.1 ± 5.8	16.7	22.2	25.0	19.4	16.7	20.0 ± 3.6	19.4	25.0	30.6	19.4	16.7	22.2 ± 5.6	21.1 ± 4.8
Position.AP, Position.V	16.7	22.2	38.9	27.8	11.1	23.3 ± 10.7	13.9	25.0	36.1	22.2	16.7	22.8 ± 8.7	13.9	27.8	36.1	27.8	8.3	22.8 ± 11.6	23.0 ± 9.5
Velocity.AP	11.1	19.4	22.2	27.8	16.7	19.4 ± 6.2	22.2	25.0	25.0	30.6	19.4	24.4 ± 4.1	13.9	19.4	22.2	27.8	16.7	20 ± 5.3	20.7 ± 5.4
Velocity.V	13.9	22.2	30.6	25.0	13.9	21.1 ± 7.2	13.9	25.0	30.6	25.0	13.9	21.7 ± 7.5	16.7	19.4	27.8	25.0	16.7	21.1 ± 5	20.8 ± 6.2
Velocity.AP, Velocity.V	11.1	22.2	38.9	19.4	27.8	23.9 ± 10.3	22.2	22.2	33.3	27.8	22.2	25.6 ± 5	13.9	22.2	38.9	22.2	22.2	23.9 ± 9.1	23.6 ± 7.9
Position.AP, Velocity.AP	13.9	22.2	25.0	27.8	19.4	21.7 ± 5.3	19.4	25.0	25.0	22.2	22.2	22.8 ± 2.3	22.2	22.2	33.3	19.4	13.9	22.2 ± 7.1	22.2 ± 4.9
Position.V, Velocity.V	11.1	22.2	27.8	27.8	22.2	22.2 ± 6.8	19.4	25.0	30.6	22.2	22.2	23.9 ± 4.2	11.1	19.4	27.8	25.0	16.7	20.0 ± 6.6	22.0 ± 5.8
Position.AP, Position.V, Velocity.AP, Velocity.V	11.1	25.0	36.1	27.8	22.2	24.4 ± 9.1	16.7	30.6	33.3	27.8	27.8	27.2 ± 6.3	13.9	27.8	38.9	19.4	16.7	23.3 ± 10.1	25.0 ± 8.2
Kinetic																			
GRF.AP	30.6	25.0	22.2	27.8	25.0	26.1 ± 3.2	27.8	30.6	27.8	36.1	22.2	28.9 ± 5	30.6	27.8	22.2	25.0	25.0	26.1 ± 3.2	27.3 ± 3.9
GRF.V	27.8	25.0	16.7	19.4	33.3	24.4 ± 6.6	27.8	22.2	13.9	22.2	38.9	25 ± 9.2	25.0	22.2	11.1	19.4	38.9	23.3 ± 10.1	24.5 ± 8.2
GRF.AP, GRF.V	25.0	27.8	16.7	16.7	36.1	24.4 ± 8.2	30.6	22.2	19.4	25.0	30.6	25.6 ± 5	27.8	27.8	13.9	27.8	33.3	26.1 ± 7.2	25.3 ± 6.5
Kinematic + Kinetic																			
Position.AP, Velocity.AP, GRF.AP	16.7	27.8	30.6	19.4	13.9	21.7 ± 7.2	27.8	36.1	22.2	38.9	33.3	31.7 ± 6.7	19.4	27.8	36.1	16.7	16.7	23.3 ± 8.5	25.6 ± 8.3
Position.V, Velocity.V, GRF.V	16.7	13.9	19.4	16.7	30.6	19.4 ± 6.51	22.2	22.2	11.1	13.9	36.1	21.1 ± 9.7	19.4	22.2	22.2	16.7	27.8	21.7 ± 4.1	20.7 ± 6.7
All: Position.AP, Position.V, Velocity.AP, Velocity.V, GRF.AP, GRF.V	19.4	25.0	22.2	16.7	22.2	21.1 ± 3.2	25.0	25.0	19.4	27.8	33.3	26.1 ± 5.0	22.2	27.8	16.7	25.0	27.8	23.9 ± 4.6	23.7 ± 4.6

## 3 Results

### 3.1 Performance evaluation

#### 3.1.1 Participants classification

Table 1 shows the prediction accuracy results of the participant classification. Regardless of the scaling approach and fold, the results showed the highest averaged prediction accuracies when kinematic data were considered and combined either alone (accuracy of 78.0% ± 9.2%) or in combination with the GRF data (accuracy of 78.3% ± 9.7%), indicating a non-advantage of adding kinetic data. The lowest averaged prediction accuracy was detected when kinetic data, particularly GRF.AP were considered (accuracy of 40.6% ± 4.8%). The combination of kinetic data resulted in an accuracy of 57.6% ± 10.6%, which was markedly lower than the accuracy achieved using combined kinematic data. The highest prediction accuracies were achieved in the kinematic combination when the classification models (i.e., SVMs) were trained with sCIL and DL data (i.e., fold 1 and 4) with an accuracy ranging between 77.8% and 91.9% across the three scaling approaches, resulting in an averaged accuracy of 85.6% ± 5.4%.

When considering the scaling approach, the highest prediction accuracies (average of the 4 folds) were recorded following no scaling approach with an accuracy of 81.1% ± 8.4% when considering the combination of kinematic data, and following batch scaling approach (accuracy of 80.6% ± 10.8%) when considering the combination of kinematic and kinetic data. In both combinations the lowest accuracies were achieved following the instance scaling approach (73.3% ± 7.0% and 76.1 ± 11.5, respectively).

Looking at each prediction accuracy across the different scaling approaches and fold procedures, the highest prediction accuracy was 91.1% registered following the batch scaling approach when considering combined kinematic data, either alone or in combination with the kinetic ones, and training the SVM with data from sCIL, DL, and R. The second highest accuracy of 88.9% was found following the no scaling approach when considering similar combinations and training data.

#### 3.1.2 Motor learning model classification

Table 2 shows the results of the learning-model-classification. Regardless of the scaling approach and fold procedures, the highest averaged prediction accuracy was 27.3% ± 3.9%, when GRF.AP data were considered. Similarly low accuracy rates were observed across various scaling approaches and input conditions, with prediction accuracies ranging between 8.3% (no-scale, position.AP, fold 1) and 38.9% (instance or batch scaling, GRF.V, and fold 5).

### 3.2 Explainability evaluation

The model explainability results obtained by LRP were evaluated across three combinations of trajectory-related inputs (position.AP/ position.V, velocity.AP/ velocity.V, GRF.AP/GRF.V) and scaling approaches (no scaling, batch scaling, instance scaling) for the participant classification task. The results for the motor learning models classification task were not evaluated because the prediction accuracy indicated that the models likely were not able to identify relevant features for the classification task.

**FIGURE 3:**
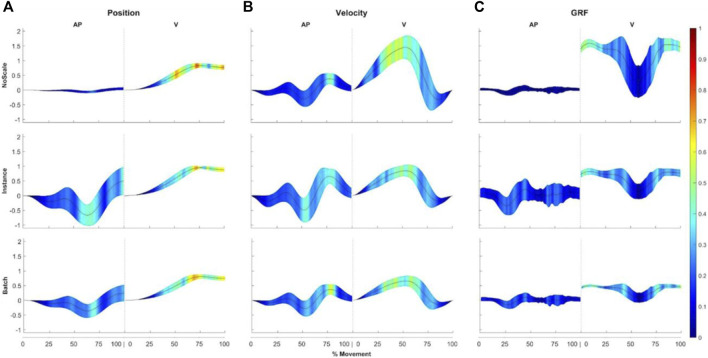
Explainability results obtained through LRP for three input combinations: **(A)** position.AP, position.V, **(B)** velocity.AP, velocity.V, and **(C)** GRF.AP, GRF.V. Each across three different scaling approaches: (top) no scale, (middle) instance, and (bottom) batch. The average input vectors (+/- one standard deviation) with aggregated colour-coded LRP relevance scores across all test samples are presented. For visualisation, input values neutral to the classification task are shown in dark blue colour, while red colour indicates input values relevant for the classification task.

Figure 3 provides an overview of which input values are relevant for discriminating between movement techniques of different participants, presenting the average input vectors with aggregated colour-coded LRP relevance scores across all test samples for the input combinations: position.AP/ position.V (Figure 3A), velocity.AP/ velocity.V (Figure 3B), and GRF.AP/GRF.V (Figure 3C). The highest LRP relevance values are observed between 40%–100% (position.V), 30%–60% (velocity.V), and 0%–15% and 80%–100% (GRF.V) of the snatch movement techniques. Across all input combinations and scaling approaches, the aggregated average LRP relevance values for the vertical input trajectories exceed those for the horizontal trajectories. This trend is particularly pronounced for the GRFs, with a notable scarcity of relevant ranges for the GRF.AP across the three scaling approaches.

While the regions with the highest LRP relevance remain consistent across the three scaling approaches, notable differences arise due to the scaling approach. This effect is most evident for the position.AP trajectory. In the no-scaling approach, the position.AP exhibits a reduced number of relevant regions for participant classification by the SVM compared to position.V. However, with instance scaling, a relevant region emerges in position.AP. Furthermore, there is a pronounced emphasis on the movement endpoint, particularly noticeable with position.V in the absence of scaling, but this effect is mitigated by batch and instance scaling. A similar trend is observed with GRF.V, albeit less prominently.

## 4 Discussion

Machine learning-based analyses of movement techniques have been recommended in the context of learning an Olympic weightlifting technique (Ammar et al., 2024a; Ammar et al., 2025), such as classification based on participant *versus* motor learning models to substantiate the individuality hypothesis in whole-body movement techniques. The employed SVM in the present study aimed at evaluating the individuality hypothesis at the very early phase of acquisition, through the classification of participants and motor learning related snatch patterns performed by novices.

The main findings revealed a superior prediction accuracy for the participant compared to the motor learning model classification. The prediction accuracy for the participant classification across all scaling approaches reached 78% for the averaged values and 91% following specific fold and input combination procedures, markedly exceeding the zero-rule baseline of 6.7% (1/15 participants). In comparison, the prediction accuracy for motor learning model classification was 27.3% for the averaged values, not heavily exceeding the zero-rule baseline of 25% (1/4 motor learning models), and 38% following specific fold and input combination procedures. This discrepancy underscores the inadequacy of motor learning model classification based on the short-term effects of single training bouts, which performs no better than the zero-rule baseline, in comparison to the strong capability to classify individual participants. This result supports the persistence of individual snatch techniques of novice participants across different motor learning models already at the very beginning of the acquisition phase. The present results provide specific evidence for the “individuality” assumption of whole-body movement techniques in the domain of Olympic weightlifting. The explainability results obtained through LRP indicate that the participant classification depends often on multiple features, with emphasis on near maxima or inflection points of the curve as well as on the start or end of movements (Figure 3) where variability is more prevalent, especially among novices (Liu et al., 2018). From a signal theoretical point of view these areas are either connected with a change of sign (zero crossing) or are associated with maxima in their next derivative that contains the most information of a signal and most probably indicates the expression of the most individual features.

The present findings contribute additional evidence to the growing body of research supporting the individuality of movement techniques, demonstrating that this individuality is evident not only at the end of a learning process (Schöllhorn and Bauer, 1998a; Schöllhorn et al., 2001; Horst et al., 2017b; Hoitz et al., 2021; Horst et al., 2023b) but also from the beginning (i.e., novices). The findings of this study align with the sport science training principle of ‘individuality’ proposed by Matveev, (1966). This principle emphasises the importance of considering individual differences among athletes throughout the training process, regardless of their performance level. In comparison, our findings challenge the training principle of increasing individualisation advocated by Harre (1975) (Martin et al., 1991; Schöllhorn et al., 2002a), which suggests that individual differences among athletes should only be taken into account in advanced performance level athletes.

The need to consider individuality of movement techniques throughout the motor learning process (i.e., during acquisition, stabilisation, and refinement phase) is further corroborated by studies that attempted to evaluate the individuality of movement techniques by assessing commonalities across diverse movement techniques (Horst et al., 2020; Burdack et al., 2023). Particularly, focusing on varied movement techniques within the same sport domain (i.e., throwing), Horst et al., 2020 used an automatic classification by means of machine learning (i.e., SVMs) to identify participant- and discipline-specific throwing techniques. Furthermore, exploring different movement techniques across various domains, Burdack et al. (2023) investigated the individuality assumption in walking, running, and handwriting techniques through a person classification. Their findings indicated distinct differences between participants in terms of GRF.V in running or walking, as well as in vertical pen pressure in handwriting with F1-scores exceeding 90%. Collectively, these results from the present study and previous research provide substantial evidence for individual characteristics both within single-movement technique as well as across different movement techniques with either similar or different kinematic structures.

Prior to this study, empirical recommendations for the most effective processing (e.g., scaling approaches) and combination of input trajectories for classifying movement techniques in Olympic weightlifting were scarce. The present study represents a pioneering effort to provide insights into optimal scaling approaches and combinations of kinematic and/or kinetic input combinations for machine learning classification in this field.

The analysis of the combinations of input trajectories on the accuracy in participant classification showed that the highest prediction accuracies with SVM models were attained using kinematic data either alone (position.AP/V, velocity.AP/V) or in combination with kinetic ones (position.AP/V, velocity.AP/V, GRF.AP/V), achieving an average accuracy of 78%, in both cases. These findings suggest that adding kinetic GRF data does not confer an advantage in terms of prediction accuracy in the studied movement technique. The exclusive use of kinetic data resulted in a lower average accuracy of 57.6%. The lower prediction accuracy of the kinetic data compared to the kinematic data, coupled with the lack of improvement in accuracy despite the inclusion of kinetic data alongside kinematic data, might be attributable to the processing approach used for the kinetic data. The data segmentation relied on the kinematic data of the barbell, which may have favoured kinematic variables. Furthermore, disparities in movement technique among the novice participants may have exerted a more pronounced effect on the GRF data. Specifically, some trials featured a flight phase during the snatch movement, while others did not. The presence or absence of flight phases, which affect GRF data significantly more than kinematic data (GRFs drop to zero while kinematic data continue their trajectory), poses greater challenges for participant classification. Future research could benefit from incorporating whole-body kinematic measures, such as ankle, knee, and hip angles, allowing for more detailed and accurate segmentation of the snatch movement’s phases. A more precise definition of movement phases and phase-dependent scaling of GRF data could potentially yield kinetic prediction accuracy comparable to that of the kinematic data. These observations underscore the critical role of adopting appropriate data preprocessing pipelines (i.e., segmentation) in machine learning-based research in biomechanics.

The analysis of the impact of different scaling approaches on the accuracy in participant classification, particularly with kinematic input combinations, revealed a decrease in accuracy when scaling is applied, particularly instance scaling, as opposed to classifications without scaling (81.1% ± 6.7% vs 73.3% ± 7.0%). This discrepancy remains notably for instance scaling even when considering a combination of both kinematic and kinetic inputs (76.1% ± 11.5%), where the accuracy rates for classifications without scaling and batch scaling stood exceeding 78%, with slight advantage of batch scaling (≈81%). These results suggest that the no scaling approach generally showed better performance, particularly with mixed kinematic data inputs, likely due to its less restrictive handling of variable ranges. This is a surprising finding, because scaling prior to machine learning-based classifications is generally recommended and accepted in the machine learning domain (Bishop, 2006). For example, to allow all input trajectories to have an equal share to the prediction of the machine learning models. Our results indicate for the position, velocity, and GRF trajectories that the AP trajectories are hardly relevant for the decision of the SVM compared to V trajectories if the data is not scaled (Figure 3). Indeed, the LRP relevance scores indicated that the vertical trajectories (position.V and GRF.V) consistently showed higher relevance values, particularly at key phases of the movement (e.g., 40%–100% for position.V and 80%–100% for GRF.V). This suggests that these phases, contain critical information for distinguishing individual movement techniques. Additionally, the scaling approach influenced the distribution of relevance scores, with instance scaling revealing relevant regions in position.AP that were not evident with no scaling. However, in particular for the position and GRF trajectories, we can observe that the start and the end phase of the movement have higher LRP relevance scores when input trajectories are not scaled prior to the SVM classification (Figure 3). This could indicate that differences in the start or end position are taken more into account by the SVM models. This may be helpful for differentiating participants (and have a positive effect on the prediction accuracy), but for classification tasks that are evaluated across participants, a stronger integration of these characteristics (which are more related to body size rather than to movement technique) may be disadvantageous. It is important to note that the input trajectories in our study were already normalised to body height and body weight. This normalisation implies that differences in amplitude range between trajectories were already mitigated. Further research is necessary to explore this aspect in depth and provide recommendations for scaling methods that consider not only classification performance but also the prevention of biases. For instance, Burdack et al. (2020) indicated a notable enhancement in classification accuracy when using advanced preprocessing pipelines prior to the classification of gait data (Burdack et al., 2020). The study demonstrated that while weight normalisation and the number of data points in time normalisation had limited effects, filtering GRF data and employing data reduction techniques, such as Principal Components Analysis, significantly improved the prediction performance of machine learning-based approaches. The extent to which these domain-specific recommendations for common data preprocessing methods in human gait are applicable to sports movements, such as strength-coordination exercises, warrants further investigation.

Overall, the present results demonstrate that the movement techniques of the individual participants exhibit a high degree of uniqueness even at the beginning of the acquisition phase in Olympic weightlifting, and that these individual movement techniques are persistent across various learning bouts with different motor learning models. However, the preliminary nature of these findings and caution against their generalisability needs to be considered. This relates, among other factors, to the following considerations:

(1) Since the present analysis was conducted using data gathered only from a standard empty barbell weighing 20 kg, these results need to be verified using higher barbell load commonly used during weightlifting training.
(2) We acknowledge the lack of kinematic data related to the athletes’ complete gestures as a main limitation of our study. Analyzing the participants’ complete gesture, in addition to the weightlifting bar, would provide deeper insights into the contributions of the different segments to the movement as well as the ratios of force and speed deployed by each subject in each technique. This would offer a more comprehensive understanding of the individuality in motor learning.
(3) The lack of perfect classification accuracy (approximately 20% misclassification) warrants further investigation in future research. Possible factors contributing to this include increased movement variability in absolute novices, a limited amount of data per participant, simple data processing (no reduction techniques, no hyperparameter tuning) and classification (linear SVMs) pipelines, or the individual variability in response to the barbell weighing of 20 kg and the motor learning models. This also raises questions about the individual’s sensitivity to specific motor learning interventions and the origins of these sensitivities.
(4) In particular, the persistence of individual movement techniques in motor learning interventions needs to be investigated in more detail. Since the present analysis encompassed only four learning bouts, each comprising 36 trials according to another motor learning model, these results need to be verified over longer time periods and controlled for differing effects between motor learning models. The variance across folds in the participant classification suggests differing effects among various motor learning models. Interestingly, within the kinematics input combination, the present findings showed their highest prediction accuracies when trained with data after the sCIL and DL training bouts (i.e., the cases of folds 1 and 4). These results suggest varying impacts of different motor learning models, particularly those that induce variability, such as sCIL and DL. However, further research is needed to determine whether the robustness and accuracy of the SVM models primarily depend on the variability of the motor learning models (either inter- or intra-variability), or if it is simply a result of a specific cross-validation procedure. A question to be clarified will be, how the variability introduced by different motor learning methods, e.g., serial or random and within or between motor programs in CIL or the size of execution-to-execution differences in DL, will affect movement variability during the learning bouts and how this will shape the long-term development of a participant’s movement technique.
(5) The uniqueness and persistence of individual movement techniques also need to be investigated in more experienced populations during the stabilisation and refinement phases of motor learning, in Olympic weightlifting.
(6) The present finding is specific to machine learning-based studies that utilise a similar data preprocessing approach and must be validated by further strength-coordination-based research employing different preprocessing methods. This future research needs to explore explainable artificial intelligence (e.g., LRP) in more detail.

Regardless of the large-scale tasks at hand, our findings encourage the applicability and efficacy of machine learning-based classifications (i.e., using SVMs) of biomechanical data in the exploration of complex human movement techniques and the identification of athlete-specific similarities in weightlifting-movement patterns across different motor learning models, especially among novice athletes. Such insights advocate for a tailored approach to data preprocessing and combinations of input trajectories to enhance model performance and reliability in identifying participant-specific movement techniques.

## 5 Conclusion

This research reinforces the feasibility of employing machine learning classification for more comprehensive analyses of biomechanical technique analysis especially in the context of movements that combine strength and coordination. The study provides additional evidence supporting the concept of the individuality of whole-body movements, highlighting its uniqueness and persistence aspects, specifically within the context of weightlifting and strength-coordination activities that engage the whole-body motor system. Notably, the study reveals that unique movement patterns can be discerned even among novice practitioners and persist post-training bouts, irrespective of the training method employed. Additional research is needed to determine whether the observed individuality is consistent across different levels of expertise, stages of motor learning, and sports disciplines with varying kinematic structures. Such research could also elucidate how coordination, strength, and endurance (each operating on distinct temporal scales) influence the long-term adaptation processes of individual movement techniques. Future research in this area should be conducted with caution, emphasizing the critical importance of the appropriate selection of input trajectories and data scaling for machine learning classifications. In more general terms, the results underline the need for a more thorough understanding of the time-dependent changes in individual living systems based on a nonclassical statistical approach as it is associated with the machine learning approach.

## Data Availability

The raw data supporting the conclusions of this article will be made available by the authors, without undue reservation.
